# Cost Effectiveness of Bosentan for Pulmonary Arterial Hypertension: A Systematic Review

**DOI:** 10.1155/2018/1015239

**Published:** 2018-11-18

**Authors:** Ruxu You, Xinyu Qian, Weijing Tang, Tian Xie, Fang Zeng, Jun Chen, Yu Zhang, Jinyu Liu

**Affiliations:** ^1^Department of Pharmacy, Union Hospital, Tongji Medical College, Huazhong University of Science and Technology, Wuhan, Hubei, China; ^2^Saw Swee Hock School of Public Health, National University of Singapore, Singapore; ^3^Department of Cardiology, Union Hospital, Tongji Medical College, Huazhong University of Science and Technology, Wuhan, Hubei, China; ^4^Department of Pharmacy, Tongji Hospital, Tongji Medical College, Huazhong University of Science and Technology, Wuhan, Hubei, China

## Abstract

**Objectives:**

Although many studies have reported on the cost-effectiveness of bosentan for treating pulmonary arterial hypertension (PAH), a systematic review of economic evaluations of bosentan is currently lacking. Objective evaluation of current pharmacoeconomic evidence can assist decision makers in determining the appropriate place in therapy of a new medication.

**Methods:**

Systematic literature searches were conducted in English-language databases (MEDLINE, EMBASE, EconLit databases, and the Cochrane Library) and Chinese-language databases (China National Knowledge Infrastructure, WanFang Data, and Chongqing VIP) to identify studies assessing the cost-effectiveness of bosentan for PAH treatments.

**Results:**

A total of 8 published studies were selected for inclusion. Among them were two studies comparing bosentan with epoprostenol and treprostinil. Both results indicated that bosentan was more cost-effective than epoprostenol, while the results of bosentan and treprostinil were not consistent. Four studies compared bosentan with other endothelin receptor antagonists, which indicated ambrisentan might be the drug of choice for its economic advantages and improved safety profile. Only two economic evaluations provided data to compare bosentan versus sildenafil, and the results favored the use of sildenafil in PAH patients. Four studies compared bosentan with conventional, supportive, or palliative therapy, and whether bosentan was cost-effective was uncertain.

**Conclusions:**

Bosentan may represent a more cost-effective option compared with epoprostenol and conventional or palliative therapy. There was unanimous agreement that bosentan was not a cost-effective front-line therapy compared with sildenafil and other endothelin receptor antagonists. However, high-quality cost-effectiveness analyses that utilize long-term follow-up data and have no conflicts of interest are still needed.

## 1. Introduction

Pulmonary arterial hypertension (PAH) is a relatively rare but life-threatening disease characterized by elevated arterial blood pressure in the pulmonary circulation that when left untreated results in right ventricular failure and death. The diagnosis is based on pressure measurements obtained by right heart catheterization and is defined as a mean pulmonary artery pressure of at least 25 mmHg, a pulmonary artery wedge pressure of not more than 15 mmHg and a pulmonary vascular resistance (PVR) of at least 3 Wood units [1]. The pathological changes of PAH include lesions in distal pulmonary arteries, medial hypertrophy, intimal proliferative and fibrotic changes, and adventitial thickening with perivascular inflammatory infiltrates. Vasoconstriction, endothelial dysfunction, dysregulated smooth muscle cell growth, inflammation, and thrombosis are contributory mechanisms to the disease progression [2]. A modified New York Heart Association (NYHA) functional classification system was adopted by the World Health Organization (WHO) in 1998 to facilitate evaluation of patients with PAH. Patients may have functional class (FC) I through IV, with increasing numbers reflecting increased severity.

Although PAH affects males and females of all ethnicities and ages, the disease is more common in women aged between 20 and 40 years old [3]. The prevalence of PAH has been reported to be between 15 and 50 cases per million population [4]. Currently, there is no cure for PAH, but the overall median survival rates have improved dramatically over the past years (from 2.8 to 7 years in the aforementioned American registry) [5, 6], presumably due to a combination of significant advances in treatment strategies and patient-support strategies.

Throughout the past 20 years, numerous specific pharmacological agents have been approved for the treatment of PAH, including prostacyclin pathway agonists (intravenous prostacyclin, synthetic analogs of prostacyclin, and nonprostanoid prostacyclin receptor agonists), endothelin receptor antagonists (ERAs), phosphodiesterase type-5 inhibitors (PDE-5Is), and the first soluble guanylate cyclase (sGC) stimulator (riociguat) [7]. As more novel therapies for PAH enter the market, it is necessary to evaluate their impacts on both economic and long-term health outcomes. Considering the limited availability of healthcare in the management of PAH, health technology assessment is increasingly important to determine whether treatments represent good value for money, as PAH is not only associated with morbidity, mortality, and overall reduced quality of life but also leads to increased healthcare expenditure [8].

Bosentan is a dual endothelin receptor antagonist and the first oral agent available in China for the treatment of PAH. Since the development of bosentan, the number of papers and articles focused on its efficacy, short- and long-term costs, and cost-effectiveness has massively increased, which have provided scientific evidence for the deeper understanding of the therapy. Despite the potential benefit of the targeted agent in the treatment of PAH, its application is discussed controversially due to their high prices. Hence, it is necessary to assess the economic impact of the use of these agents in PAH.

The objective of this article is, therefore, to review and assess the economic evidence of treatments with targeted agent bosentan in PAH. The review was also conducted to provide insight into key drivers of cost-effectiveness ratios and help healthcare decision-makers, patients, and health systems leaders make well-informed decisions.

## 2. Methods

The PRISMA (Preferred Reporting Items for Systematic Reviews and Meta-Analyses) guidelines by Moher et al. were followed for review and reporting procedures [9].

### 2.1. Eligibility Criteria

To be included in this systematic review, the articles had to meet the following criteria: (1) identified as a full economic evaluation, examined costs and their consequences, and reported incremental cost-effectiveness ratios (ICERs) or incremental cost-utility ratios (ICURs); (2) they included the bosentan intervention, regardless of monotherapy or combinations therapy; and (3) they were available in complete full-text format. Articles were excluded if they were systematic reviews, expert opinions, comments (commentary), methodological article, or conference abstracts and proceedings.

### 2.2. Literature Search

We conducted a systematic literature search to identify all relevant studies estimating the cost-effectiveness of PAH therapies published between 1 January 2000 and 30 June 2017. The following databases were searched: MEDLINE (PubMed), EMBASE (Ovid), EconLit databases, and the Cochrane Library for English-language studies; and China National Knowledge Infrastructure (CNKI), Wanfang Data and Chongqing VIP (CQVIP) for Chinese-language studies. Literature search algorithm is detailed in Appendix S1.

### 2.3. Study Selection

The titles and abstracts were screened for eligibility by two independent authors. Full-text copies of all potentially relevant articles were obtained and reviewed to determine whether they met the prespecified inclusion criteria. Disagreements were resolved by consensus through discussion.

### 2.4. Data Collection

Data on study and patient characteristics as well as relevant outcomes were extracted using a standardized data extraction form, including general information for the article (e.g., authors and publication year), characteristics of the study (e.g., design and sample size), type of economic evaluation, study objective, description of the intervention and comparators, measure of benefit, cost data and respective sources, methods for dealing with uncertainty as well as cost and outcome results.

### 2.5. Quality Assessment

The quality of reporting of all included studies was appraised using the 24-items Consolidated Health Economic Evaluation Reporting Standards (CHEERS) statement. Each item in the CHEERS checklist was scored as having met the criteria in full (“1”), not at all (“0”), or not applicable (NA). Quality assessment was performed by two authors, and the remaining authors resolved conflicts through discussion and consensus. Studies with a score higher than 75% were categorized as good, studies in the range 50%–74% were categorized moderate, and studies with scores lower than 50% were categorized as low [10].

### 2.6. Data Synthesis

A narrative synthesis was used to summarize and evaluate the aims, methods, settings, and results of the studies reviewed. When possible, information was compared across studies about the modeling technique, the cost perspective, the measures of benefit used, and incremental cost-effectiveness ratios. Cost/charges data are presented in US$ for the common price year 2017 using the “CCEMG-EPPI-Centre Cost Converter” Version 1.5 [11], a web-based tool that can be used to adjust an estimate of cost expressed in one currency and price year to a target currency and/or price year.

## 3. Results

### 3.1. Studies Identified

**Figure 1:**
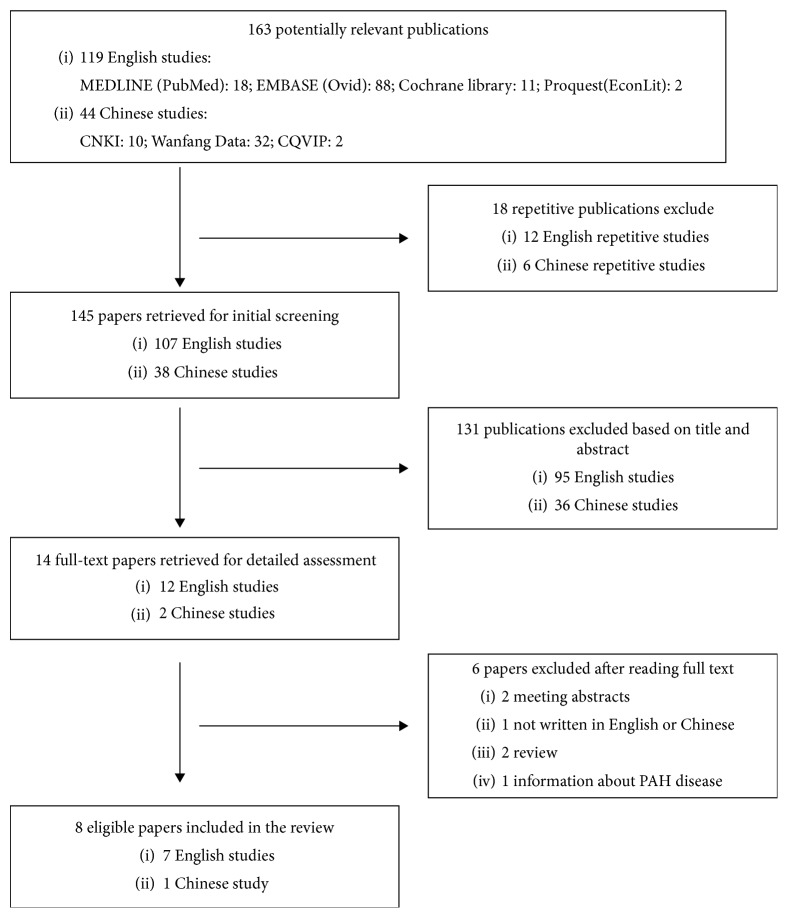
Flowchart of literature search. CNKI China National Knowledge Infrastructure database, CQVIP Chongqing VIP database, and PAH pulmonary arterial hypertension.

A total of 163 potential publications were identified with the search strategy used, including 119 English-language studies and 44 Chinese-language studies, among which 18 were duplicates and 131 were excluded after screening and analysis of titles and abstracts for not matching the eligibility criteria. A total of 8 articles were retrieved and analyzed (Figure 1).

### 3.2. Description of Identified Studies

The 8 included studies are detailed in Table 1. Two studies [12, 13] were conducted for the USA, and two for Canada [14, 15]. The remaining studies were conducted for Australia [16], UK [17], China [18], and Italy [19]. Of the 8 studies included, five used the Markov model [12–14, 17, 18], two used the Excel model [16, 19], and one used the cost-minimization analysis [15]. Five studies were conducted from the perspective of healthcare payers, of which four were performed from the perspective of public payers (e.g. Canadian Healthcare System, National Health System) [14, 15, 17, 19], while one study used a third-party payer perspective [16]. And in three studies [12, 13, 18] the perspective was not stated. The time horizons used in the EXCEL and Markov models were highly variable ranging from 3 years to a lifetime. Four studies [12, 13, 15, 19] used a shorter, 3 or 5-year time horizon, while the remaining studies [14, 16–18] chose longer modeling horizons such as 15 years.

The endothelin receptor antagonist in the included studies was bosentan, and the most frequent application for comparators was prostanoids [12, 13], ambrisentan [13–15, 19], sildenafil [13, 14], and conventional, supportive, or palliative therapy [14, 16–18]. The majority of studies reported results as ICERs. Two studies were sponsored by Actelion Pharmaceuticals [16, 17], two were funded by GlaxoSmithKline [15, 19], one received funds from Actelion Pharmaceuticals, Encysive Pharmaceuticals, CoTherix, Gilead Sciences, United Therapeutics, and Pfizer [13], and one was funded by the Canadian Agency for Drugs and Technologies in Health (CADTH) [14]. Two studies [12, 18] did not disclose the source of funding.

### 3.3. Quality Assessment

Based on reporting quality assessment from the CHEERS statement, most of the studies were classified as high quality [13–17, 19] and two as moderate [12, 18].  Table 2 presented the proportion of each item in the CHEERS checklist that was reported sufficiently, partially, or not at all in the review. Two studies [12, 18] failed to report the source of funding, and no state and the conflicts of interest were given by five studies [12, 15–18]. Additionally, no studies stated the setting and location—also an item required by the checklist when reporting the background and objectives of economic evaluations. Moreover, the perspective of the study of Highland et al. [12], Garin et al. [13] and Fan et al. [18]was not stated. Reasons for the choice of time horizon were not reported in three studies [12, 13, 19]. Only one study [17] performed a subgroup analysis to assess the impacts of bosentan in iPAH and PAH-CTD. And no studies [12–19] discussed the generalizability of the results; even they reported the study findings and limitations.

### 3.4. Cost-Effectiveness Results of the Studies

The results of the included studies for the cost-effectiveness analysis are summarized in Table 3.

#### 3.4.1. Bosentan versus Prostanoids

Two studies [12, 13] conducted in the USA provided economic evaluation data for bosentan versus prostanoids (epoprostenol, treprostinil, and iloprost). Highland et al. [12] developed a Markov model to compare the cost-effectiveness of bosentan, epoprostenol, and treprostinil in treating PAH in 2003. These studies reported that bosentan was more cost-effective than either epoprostenol or treprostinil, with lower costs (a cost saving of $46417.21 or $62289.22, respectively. Adjusted to the year 2017 value) and a greater gain in quality-adjusted life-years (QALYs; 0.11 more QALYs gained) per patient. Garin et al. [13] improved the research by Highland et al. [12] in the updated Markov model in 2009. Bosentan was found to be dominant (lower costs and greater QALY) relative to other medications epoprostenol and iloprost. Additionally, in contrast to the findings by Highland et al. [12], Garin et al. [13] found that treatment with treprostinil resulted in an average annual savings of $4818.51 (adjusted to the year 2017 value) when used as an alternative to bosentan, with an ICER of $81393.84 per QALYs (adjusted to the year 2017 value) gained. The ICER of bosentan therapy was $81393.84 per QALY, adjusted to the year 2017 value, greater than the cost-effectiveness threshold of $50000 per QALY in the USA.

#### 3.4.2. Bosentan versus Other Endothelin Receptor Antagonists

Four studies [13–15, 19] compared bosentan with other available endothelin receptor antagonists, including ambrisentan and sitaxentan. Two studies [13, 14] used the cost-effectiveness analysis, while the other two used the cost-minimization analysis [15] and the budget impact analysis [19], respectively.

Coyle et al. [14] estimated that, as first-line medications, both 5 mg and 10 mg ambrisentan provided more QALYs and cost-saving values than bosentan for patients with either FCII or III PAH. Garin et al. [13] found that the cost-effective value of bosentan was similar to that of ambrisentan. However, sitaxentan, as an alternative to bosentan, its annual cost saving $525.43 per 100 patients (adjusted to the year 2017 value) and the ICER was $3283.94 per QALYs (adjusted to the year 2017 value) which is within the threshold of acceptability in the USA. In the study of Dranitsaris et al. [15], which built a population-based CMA model to evaluate 3 years pharmacotherapy, revealed that bosentan would be associated with higher costs of $14956.40 and $5786.41 (adjusted to the year 2017 value) when used as an alternative to ambrisentan or sitaxentan, respectively. Moreover, the budget impact analysis reported by Barbieri et al. [19] demonstrated that the use of ambrisentan instead of bosentan for eligible patients might result in savings of about $1.1 million (adjusted to the year 2017 value) over a 3-year time horizon in Italy.

#### 3.4.3. Bosentan versus Sildenafil

Two studies [13, 14] provided economic evaluation data for bosentan versus sildenafil in patients with PAH in the USA and Canada. Garin et al. [13] built the Markov-type model to evaluate 1-year treatment; the economic analysis and sensitivity analysis indicated that treatment with bosentan resulted in the same gain in QALYs as sildenafil, but at a higher cost. In another study [14], a cost-utility analysis suggests that sildenafil was less costly (FC II $121171.50 versus $336604.81; FC III $150057.17 versus $342153.27, adjusted to the year 2017 value) and more effective (FC II 4.663 QALYs versus 3.904 QALYs; FC III 3.284 QALYs versus 2.960 QALYs) than bosentan in PAH patients with either functional class FC II or III disease. Therefore, the results show that the initiation of treatment with sildenafil is likely the most economical option.

#### 3.4.4. Bosentan versus Conventional, Supportive or Palliative Therapy

Four studies [14, 16–18] conducted in Canada, Australia, the UK, and China compared bosentan with conventional, supportive, or palliative therapy. Wlodarczyk et al. [16] assessed an average cost of $204237.00 (adjusted to the year 2017 value) per patient for bosentan and $15918.99 (adjusted to the year 2017 value) for conventional therapy alone in Australia, and bosentan was associated with an incremental life expectancy of 3.87 years over palliative therapy, with an ICER of $48660.98 per life-year (LY) gained over a 15-year time horizon. Stevenson et al. [17] indicated that bosentan was likely to be a more potential cost-effective first-line therapy for UK patients over the lifetime with iPAH or PAH-CTD within FC III than palliative care, with less costly (IPAH $216808.07 versus $328448.05 per patient; PAH-CTD $100314.18 versus $152089.25 per patient, adjusted to the year 2017 value) and better outcomes in QALYs (IPAH 3.32 versus 2.95; PAH-CTD 1.36 versus 1.21). The study by Fan et al. [18] showed that the utility value, which represented the health-related quality of life, was 7.23 QALYs treated with bosentan therapy and 1.04 QALYs with palliative therapy, respectively. Bosentan was associated with an incremental gain of 6.19 QALYs over palliative therapy. The estimated cost per patient was $143837.35 for patients treated with bosentan and $18610.09 for those given palliative therapy, a cost increase of $125227.26 per patient. The incremental cost-utility of bosentan relative to palliative therapy was $20230.58, which was less than one half gross domestic product (GDP) in China.

In comparison, in the study of Coyle et al. [14], bosentan did not show a conclusive effect on cost-effectiveness. The study conducted in Canada showed that the ICER of bosentan versus supportive care in both patients with FC II and III diseases remained $303291.55 or $633344.58, which was higher than the willingness-to-pay threshold of $165700.08.

Cost-effectiveness analyses of bosentan versus palliative therapy also suggested that bosentan was a potentially cost-effective intervention in Australia, UK, and China, which is not consistent with the results of Highland et al. [12] in the USA.

## 4. Discussion

### 4.1. Summary of Evidence

PAH is a chronic progressive devastating disease with no cure, which may bring a significant medical and financial burden to patients' families. The aim of this systematic review of published studies was to evaluate the costs and cost-effectiveness of bosentan for PAH. We used the thresholds stated in the included studies if applicable. Otherwise, we searched the literature to identify appropriate and accepted thresholds used in relevant countries to determine if the ICERs of bosentan was below such criteria, and in turn to evaluate whether they appeared to provide good value or money for PAH.

In our analysis, two other Markov model-based cost-effectiveness analyses of bosentan, epoprostenol, and treprostinil in treating PAH was published in the USA [12, 13]. In these two studies, both of their results indicated that bosentan was more cost-effective than epoprostenol, while the results of bosentan and treprostinil were not consistent. The differences between bosentan and treprostinil might be ascribed to different methodological approaches, such as the model structure, time horizon, the measurement of costs, and health utilities.

According to the four model results, in PAH patients for whom an ERA was the preferred agent, ambrisentan might be the drug of choice because of its economic advantages and improved safety profile [13, 14, 16, 19].

Two studies investigated the cost-effectiveness analysis of bosentan versus sildenafil, and sildenafil was founded to be more cost-effective, especially for adult patients with FC II and III PAH [13, 14].

Among four studies [14, 16–18] that compared bosentan with conventional, supportive, or palliative therapy, three studies [16–18] concluded that bosentan seemed to be more favorable. In comparison, Coyle et al. [14] indicated that long-term, head-to-head research must be conducted to evaluate the cost-effectiveness of bosentan and supportive care before making any recommendations in Canada. This study differed from the current analysis as it was not designed to allow a direct comparison of the cost-effectiveness of PAH treatments relative to each other, but only relative to supportive care.

### 4.2. Quality of Evidence

With regard to the quality of reporting of these economic evaluations, despite the fact that guidelines for conducting health economic evaluations have been widely available for many years, we observed that the quality of reporting was still insufficient for several articles. We hope that the availability of the CHEERS statement will lead to improvement in the reporting and hence the quality of economic evaluations of PAH.

In terms of characterizing heterogeneity, subgroups of patient's diverse baseline characteristics and other variables could potentially contribute to the variation of interpretation of our review results. In this review, only one study [17] performed a subgroup analysis of iPAH and PAH-CTD. In fact, subgroup analyses are important because some factors such as disease severity, gender, and body mass index (BMI) may also affect the prognosis of PAH and exert a direct effect on its budget to a new therapy [20, 21].

In addition, it is noteworthy that several articles included in this study receive grants from pharmaceutical companies which might cause potential bias of cost-effective evaluations. Although pharmaceutical industry-funded research could result in biases in cost-effective analyses, no guidance currently exists on how to evaluate this bias.

### 4.3. Key Drivers of Cost-Effectiveness

In line with prior studies, some key drivers of cost-effectiveness were found in our review. First, the consideration of the comparator is highly important. The cost-effectiveness of a drug therapy could differ according to the selected comparator. For example, bosentan was shown to be dominant relative to epoprostenol, while the cost-effectiveness was less favorable when using sildenafil or ambrisentan as the comparator. Therefore, one of the most important structural choices of a cost-effectiveness analysis is the comparator choice.

On the contrary, the included analyses were largely country-specific since healthcare systems and reimbursement policies could differ between countries and therefore have a significant influence on the results and final conclusions of economic evaluations. Future assessment is needed to use approaches such as alternative ways to specifying multilevel models for analyzing cost-effectiveness data and identification of a range of appropriate covariates to handle assumptions and uncertainties in economic evaluation results, which would improve the generalizability and transferability of studies across settings [22].

### 4.4. Strengths and Limitations

To the best of our knowledge, this is the first systematic review of published studies to examine the cost-saving or cost-effective properties of bosentan for PAH patients. Contrary to previous systematic and narrative reviews which were outdated or restricted to a specific comparator, this is the most comprehensive review incorporating economic evaluations over an extended period of time with quality assessed using a validated instrument.

Although this review was conducted using explicit, systematic methods that were selected to minimize bias, several limitations which affect the conclusion should be taken into consideration when interpreting the results.

First, given the disparity in the methods used across existing economic evaluations, it is extremely difficult to synthesize the studies into a coherent whole. Studies would have to be adjusted to achieve standardized results, but this is rarely achievable because of the diverse nature of the elements considered, including different types of models, perspectives, time horizons, and healthcare systems. Such difference was likely to have important impacts on model inputs such as costs and health utilities. Therefore, we summarized the evidence qualitatively, and then the results should be interpreted with caution.

Second, the trial populations used in the pharmacoeconomic models may not represent the entire PAH patients in the real-world setting. The prevalence of mortalities and comorbidities seems to be relatively low in most studies. For example, Fan et al. developed the Markov model, based on Australia and New Zealand patient population, to analyze the annual mortality rates in Chinese PAH patients, which might have lead to an inaccurate extrapolation of results, given the study populations.

Third, some relevant studies may have been overlooked in our review, especially those that were not published in the English or Chinese language. Similarly, we did not formally assess potential publication bias that may have occurred due to the lack of inclusion of unpublished studies (e.g., industry-sponsored evaluations), which may have had unfavorable findings.

## 5. Conclusions

Evidence produced by economic evaluations in general, and in the PAH field in particular, has the potential of informing clinical and reimbursement decision-making. Based on the available evidence, we conclude that the administration of bosentan for PAH appears to be a more cost-effective alternative compared with epoprostenol and conventional or palliative therapy. There was unanimous agreement that bosentan was not a cost-effective front-line therapy compared with sildenafil and other endothelin receptor antagonists. Future research investigating ways to improve the quality of reporting of economic evaluations is therefore warranted.

## Figures and Tables

**Table 1:** General characteristics of the included studies.

References	Year published, country	Perspective	Model type	Target population	Treatment	Comparator	Cost components	Time horizon	Discount rate (%)	Source of effectiveness and safety data
Highland et al. [12]	2003, USA	Unclear	Markov model	Patients with PAH	Bosentan	Epoprostenol, treprostinil	Drug, diluent, per diem, hospitalization, home health, Hickman catheter, liver function	One year	NA	Three studies
Garin et al. [13]	2009, USA	Unclear	Markov model	Patients with FC III and IV PAH	Bosentan	Epoprostenol, treprostinil, iloprost, sitaxentan, ambrisentan, sildenafil	Drug, per diem, pain medications, hospitalization/clinic visit, intravenous line infections, laboratory tests	One year	NA	Two RCTs
Coyle et al. [14]	2016, Canada	Healthcare system	Markov model	Patients with FC II and III PAH	Bosentan	Ambrisentan, sildenafil, tadalafil, supportive care	Drugs, monitoring/therapeutic procedures (includes liver function tests, pregnancy test, echocardiograms, renal function, and blood work), hospital/ER/clinic visits (includes general practitioner visits, specialist visits, nurse visits, hospitalizations, emergency room visits, therapeutic procedures), Supportive care drugs	Lifetime	5	A network meta-analysis
Dranitsaris et al. [15]	2009, Canada	Canadian healthcare system	Cost-minimization analysis (CMA)	Patients with FC II and III PAH	Bosentan	Ambrisentan, sitaxentan, sildenafil	Drug acquisition, medical consultations and visits, laboratory and diagnostic procedures, functional studies, other healthcare-related resources, alternative pharmacotherapy	3 years	3	Nine placebo-controlled trials
Wlodarczyk et al. [16]	2006, Australia	A healthcare payer perspective	An excel model	Patients with iPAH	Bosentan	Conventional therapy	Exercise test, lung function, chest x-ray, echocardiogram, electrocardiogram, blood tests, specialist, total medical	15years	5	Two aforementioned pivotal clinical trials and their long-term open-label extensions
Stevenson et al. [17]	2009, UK	National Health Service	Markov model	Patients with iPAH or PAH-CTD of FC III	Bosentan	Palliative therapy	Drug acquisition, home delivery, palliative care	Lifetime	3.5	Two RCTs
Fan et al. [18]	2016, China	Unclear	Markov model	Patients with PAH	Bosentan	Palliative therapy	Drugs, monitoring/therapeutic procedures	Lifetime	3.5	Patient registration and follow-up data for charity project
Barbieri et al. [19]	2014, Italy	National Health System	An excel model	Patients with FC II and III PAH	Bosentan	Ambrisentan	Drug acquisition cost, direct medical costs (includes visits to professionals, laboratory tests, concomitant medications, hospitalizations)	3 years	Unclear	Two separate double-blind studies

*Note.* PAH: pulmonary arterial hypertension; FC: functional class; NA: not applicable; RCT: randomized controlled trial; CTD: connective tissue disease; CMA: cost minimization analysis.

**Table 2:** Quality of the economic evaluations (as assessed by the CHEERS statement).

Item No.	Section/item	1	2	3	4	5	6	7	8
		Highland KB et al. [12]	Garin MC et al. [13]	Coyle K et al. [14]	Dranitsaris G et al. [15]	Wlodarczyk JH et al. [16]	Stevenson MD et al. [17]	Fan et al. [18]	Barbieri M et al. [19]
1	Title	1	1	1	1	1	1	1	1
2	Abstract	1	1	1	1	1	1	1	1
3	Background and objectives	1	1	1	1	1	1	1	1
4	Target population and subgroups	1	1	1	1	1	1	1	1
5	Setting and location	0	0	0	0	0	0	0	0
6	Study perspective	0	0	1	1	1	1	0	1
7	Comparators	1	1	1	1	1	1	1	1
8	Time horizon	1	1	1	1	1	1	1	1
9	Discount rate	0	0	1	1	1	1	1	0
10	Choice of health outcomes	1	1	1	1	1	1	1	1
11	Measurement of effectiveness	1	1	1	1	1	1	1	1
12	Measurement and valuation of preference-based outcomes	1	1	1	1	1	1	1	0
13	Estimating resources and costs	1	1	1	1	1	1	1	1
14	Currency, price date, and conversion	1	1	1	1	1	1	1	1
15	Choice of model	1	1	1	1	1	1	1	1
16	Assumptions	1	1	1	1	1	1	1	1
17	Analytical methods	1	1	1	1	1	1	1	1
18	Study parameters	1	1	1	1	1	1	1	1
19	Incremental costs and outcomes	1	1	1	1	1	1	1	1
20	Characterizing uncertainty	1	1	1	1	1	1	1	1
21	Characterizing heterogeneity	0	0	0	0	0	1	0	0
22	Study findings, limitations, generalizability, and current knowledge	0	0	0	0	0	0	0	0
23	Source of funding	0	1	1	1	1	1	0	1
24	Conflicts of interest	0	1	1	0	0	0	0	1
Overall quality	Moderate	Good	Good	Good	Good	Good	Moderate	Good	

*Note.* “1” meets the quality assessment criteria; “0” does not fully conform to the quality assessment criteria; CHEERS: Consolidated Health Economic Evaluation Reporting Standards.

**Table 3:** Overview of economic evaluation outcomes of included studies.

References	Comparison	Effectiveness/benefits	Costs (original currency; mean)	Costs (2017 US$; mean)	ICER (2017 US$ per QALY)	Threshold of ICER (per QALY)	Sensitivity or uncertainty analysis
Highland et al. [12]	(1) Bosentan vs. epoprostenol	Incremental effectiveness: 11 QALYs per 100 patients	Incremental costs: $3631900 per 100 patients/yr	Incremental costs: $4641721.88 per 100 patients/yr	Dominating	NA	Sensitivity analyses: results robust.
	(2) Bosentan vs. treprostinil	Incremental effectiveness: 11 QALYs per 100 patients	Incremental costs: $4873800 per 100 patients/yr	Incremental costs: $6228922.62 per 100 patients/yr	Dominating	NA	

Garin et al. [13]	(1) Bosentan vs. epoprostenol	Incremental effectiveness: 5.77 QALYs per 100 patients	Incremental costs: $408213 per 100 patients/yr	Incremental costs: $452508.19 per 100 patients/yr	Dominating	NA	Sensitivity analyses had minimal impact on these results.
	(2) Bosentan vs. treprostinil	Incremental effectiveness: 5.92 QALYs per 100 patients	Incremental costs: $434684 per 100 patients/yr	Incremental costs: $481851.56 per 100 patients/yr	$81393.84	$50000	
	(3) Bosentan vs. iloprost	Incremental effectiveness: 3.09 QALYs per 100 patients	Incremental costs: $3466486 per 100 patients/yr	Incremental costs: $3842634.40 per 100 patients/yr	Dominating	NA	
	(4) Bosentan vs. Sitaxentan	Incremental effectiveness: 0.16 QALYs per 100 patients	Incremental costs: $474 per 100 patients/yr	Incremental costs: $525.43 per 100 patients/yr	$3283.94	$50000	
	(5) Bosentan vs. ambrisentan	Incremental effectiveness: 0 QALYs per 100 patients	Incremental costs: $0 per 100 patients/yr	Incremental costs: $0 per 100 patients/yr	$0	NA	
	(6) Bosentan vs. sildenafil	Incremental effectiveness: 0 QALYs per 100 patients	Incremental costs: $3153535 per 100 patients/yr	Incremental costs: $3495725.08 per 100 patients/yr	Dominated	NA	

Coyle et al. [14]	(1) Bosentan vs. ambrisentan 5mg	Patients with FC II Incremental effectiveness: −0.73 QALYs per person (treatment, 3.904 QALYs; comparator, 4.634 QALYs)	Patients with FC II Incremental costs: Can$29095 per person (treatment, Can$406282; comparator, Can$377187)	Patients with FC II Incremental costs: $24105.22 per person (treatment, $336604.81; comparator, $312499.59)	Dominated	NA	Extensive sensitivity analyses: results robust. Probabilistic sensitivity analysis: results robust.
		Patients with FC III Incremental effectiveness: −0.22 QALYs per person (treatment, 2.960 QALYs; comparator, 3.180 QALYs)	Patients with FC III Incremental costs: Can$61406 per person (treatment, Can$412979; comparator, Can$351573)	Patients with FC III Incremental costs: $50874.90 per person (treatment, $342153.27; comparator, $291278.38)	Dominated	NA	
	(2) Bosentan vs. ambrisentan 10mg	Patients with FC II Incremental effectiveness: −0.313 QALYs per person (treatment, 3.904 QALYs; comparator, 4.217 QALYs)	Patients with FC II incremental costs: Can$28759 per person (treatment, Can$406282 comparator, Can$377523)	Patients with FC III Incremental costs: $23826.84 per person (treatment, $336604.81; comparator, $312777.96)	Dominated	NA	
		Patients with FC III Incremental effectiveness: −0.083 QALYs per person (treatment, 2.960 QALYs; comparator, 3.043 QALYs)	Patients with FC III Incremental costs: Can$36095 per person (treatment, Can$412979; comparator, Can$376884)	Patients with FC III Incremental costs: $29904.7 per person (treatment, $342153.27; comparator, $312248.55)	Dominated	NA	
	(3) Bosentan vs. sildenafil	Patients with FC II incremental effectiveness: −0.7593 QALYs per person (treatment, 3.904 QALYs; comparator, 4.663 QALYs)	Patients with FC II incremental costs: Can$260028 per person (treatment, Can$406282 comparator, Can$146254)	Patients with FC II incremental costs: $215433.31 per person (treatment, $336604.81; comparator, $121171.50)	Dominated	NA	
		Patients with FC III Incremental effectiveness: −0.324 QALYs per person (treatment, 2.960 QALYs; comparator, 3.284 QALYs)	Patients with FC III Incremental costs: Can$231860 per person (treatment, Can$412979; comparator, Can$181119)	Patients with FC III Incremental costs: $192096.11 per person (treatment, $342153.27; comparator, $150057.17)	Dominated	NA	
	(4) Bosentan vs. tadalafil	Patients with FC II Incremental effectiveness: −0.098 QALYs per person (treatment, 3.904 QALYs; comparator, 4.002 QALYs)	Patients with FC II Incremental costs: Can$253037 per person (treatment, Can$406282 comparator, Can$153245)	Patients with FC II Incremental costs: $209641.26 per person (treatment, $336604.81; comparator, $126963.55)	Dominated	NA	
		Patients with FC III Incremental effectiveness: −0.053 QALYs per person (treatment, 2.960 QALYs; comparator, 3.013 QALYs)	Patients with FC III Incremental costs: Can$212395 per person (treatment, Can$412979; comparator, Can$200584)	Patients with FC III Incremental costs: $175969.35 per person (treatment, $342153.27; comparator, $166183.93)	Dominated	NA	
	(5) Bosentan vs. supportive care	Patients with FC II Incremental effectiveness: 0.686 QALYs per person (treatment, 3.904 QALYs; comparator, 3.128 QALYs)	Patients with FCII Incremental costs: Can$251126 per person (treatment, Can$406282 comparator, Can$155156)	Patients with FCII Incremental costs: $208058.00 per person (treatment, $336604.81; comparator, $128815.82)	$303291.55	$165700.08 (Can$200000)	
		Patients with FC III Incremental effectiveness: 0.273 QALYs per person (treatment, 2.960 QALYs; comparator, 2.687 QALYs)	Patients with FC III Incremental costs: Can$208694 per person (treatment, Can$412979; comparator, Can$204285)	Patients with FC III Incremental costs: $172903.07 per person (treatment, $342153.27; comparator, $169250.2)	$633344.58	$165700.08 (Can$200000)	

Dranitsaris et al. [15]	(1) Bosentan vs. ambrisentan	NA	Incremental costs: Can$16302 per patient (treatment, Can$164745; comparator, Can$148443)	Incremental costs: $14956.40 per patient (treatment, $151146.60; comparator, $136190.20)	NA	NA	One-way sensitivity analysis: results sensitive to sildenafil dose, ambrisentan daily drug cost, and bosentan daily drug cost.
	(2) Bosentan vs. sitaxentan	NA	Incremental costs: Can$6307 per patient (treatment, Can$164745; comparator, Can$158444)	Incremental costs: $5786.41 per patient (treatment, $151146.60; comparator, $145365.70)	NA	NA	
	(3) Bosentan vs. sildenafil	NA	Incremental costs: Can$116394 per patient (treatment, Can$164745; comparator, Can$48351)	Incremental costs: $106786.59 per patient (treatment, $151146.60; comparator, $44360.01)	NA	NA	
Wlodarczyk et al. [16]	Bosentan vs. conventional care	At 5years incremental effectiveness: 1.39 life expectancy	At 5years incremental costs: A$116929 for each patient	At 5years incremental costs: $101787.70 for each patient	$73228.56	$41928.72 (A$60000)	One-way sensitivity analysis: removing the PBS continuation rules from the model, halving of the annual mortality rate in patients treated with conventional therapy, and changing mortality and hospitalization RR affected the results.
		At 10years incremental effectiveness: 2.93 life expectancy	At 10years incremental costs: A$181808 for each patient	At 10years incremental costs: $158265.43 for each patient	$54015.51	$41928.72 (A$60000)	
		At 15years incremental effectiveness: 3.87 life expectancy	At 15years incremental costs: A$216331 for each patient	At 15years incremental costs: $188318.00 for each patient	$48660.98	$41928.72 (A$60000)	

Stevenson et al. [17]	Bosentan vs. palliative therapy	Patients with iPAH Incremental effectiveness: 0.37 QALYs per patient (treatment, 3.32 QALYs; comparator, 2.95 QALYs)	Patients with iPAH Incremental costs: £69000 per patient (treatment, £134000; comparator, £203000)	Patients with iPAH Incremental costs: $111639.98 per patient (treatment, $216808.07; comparator, $328448.05)	Dominating	NA	The results were similar in both the deterministic and probabilistic analyses.
		Patients with PAH-CTD Incremental effectiveness: 0.15 QALYs per patient (treatment, 1.36 QALYs; comparator, 1.21 QALYs)	Patients with PAH-CTD Incremental costs: £32000 per patient (treatment, £62000; comparator, £94000)	Patients with PAH-CTD Incremental costs: $51775.06 per patient (treatment, $100314.18; comparator, $152089.25)	Dominating	NA	

Fan et al. [18]	Bosentan vs. palliative therapy	Incremental effectiveness: 6.19 QALYs per person (treatment, 7.23 QALYs; comparator, 1.04 QALYs)	Incremental costs: ¥439046.77 per patient (treatment, ¥504293.75; comparator, ¥65246.98)	Incremental costs: $125227.26 per patient (treatment, $143837.35; comparator, $18610.09)	$20230.58	$39815.46 (¥139593)	Sensitivity analyses: results robust.

Barbieri et al. [19]	Bosentan vs. ambrisentan	NA	Incremental costs: €1112145 (treatment, €87594291; comparator, €86482146)	Incremental costs: $1184990.49 (treatment, $93331717.06; comparator, $92146726.56)	NA	NA	The sensitivity analysis corroborated the base case findings.

*Note.* “Dominating” denotes bosentan treatment producing more QALYs at a lower cost, whereas “dominated” denotes bosentan producing less QALYs at a higher cost. ICER: incremental cost-effectiveness ratio; QALY: quality-adjusted life-year; yr: year; PAH: pulmonary arterial hypertension; FC: functional class; NA: not applicable; CTD: connective tissue disease.

## List of abbreviations

PAH: pulmonary arterial hypertension
PVR: pulmonary vascular resistance
FC: functional class
ERAs: endothelin receptor antagonists
PDE-5Is: phosphodiesterasetype-5 inhibitors
sGC: soluble guanylate cyclase
PRISMA: Preferred Reporting Items for Systematic Reviews and Meta-Analyses
CHEERS: Consolidated Health Economic Evaluation Reporting Standards
PPP: purchasing power parities
CCEMG: Campbell and Cochrane Economics Methods Group
EPPI-Centre: Evidence for Policy and Practice Information and Coordinating Centre
ICERs: incremental cost-effectiveness ratios
CADTH: Canadian Agency for Drugs and Technologies in Health
QALYs: quality-adjusted life-years
ICER: incremental cost-effectiveness ratio
LY: life-year
GDP: gross domestic product
BMI: body mass index

## Supplementary Materials

Supplementary Materials Table S1: literature search algorithm.
