# Supplementary material for: Cost Effectiveness of Bosentan for Pulmonary Arterial Hypertension: A Systematic Review
Source: Can Respir J. 2018 Nov 18;2018:1015239. doi: 10.1155/2018/1015239 (PMC6276424; doi:10.1155/2018/1015239)
Supplement: Supplementary Materials — Table S1: literature search algorithm. [file 1015239.f1.docx]

**Cost Effectiveness of Bosentan for pulmonary arterial hypertension: A Systematic Review**

Ruxu You, Xinyu Qian, Weijing Tang, Tian Xie, Fang Zeng, Jun Chen, Yu Zhang, Jinyu Liu

**S****upplementary Information**

Table S1. Literature search algorithm

**MEDLINE (PubMed)**

| Search strategy | | Item found |
| --- | --- | --- |
| #1 | (Cost-benefit analysis[MeSH] OR (cost*[tiab] AND (benefit[tiab] OR effectiveness[tiab] OR utility[tiab]) OR (marginal[tiab] AND analys*[tiab]) OR minimization OR minimisation) OR ((economic*[tiab] OR pharmacoeconomic*[tiab]) AND (analys*[tiab] OR assessment*[tiab] OR evaluat*[tiab] OR implication[tiab] OR health[tiab]))) | 262575 |
| #2 | (Familial Primary Pulmonary Hypertension[Mesh] OR Pulmonary Arterial Hypertension[Supplementary Concept] OR Hereditary Hemorrhagic Telangiectasia-Related [Supplementary Concept] OR Dursun Syndrome [Supplementary Concept] ) OR(Hypertension, Pulmonary[Mesh] OR Familial Primary Pulmonary Hypertension[Mesh] OR Persistent Fetal Circulation Syndrome[Mesh] OR Pulmonary Hypertension, Primary, Autosomal Recessive[Supplementary Concept] OR Cirrhosis, Familial, with Pulmonary Hypertension[Supplementary Concept] OR Pulmonary edema of mountaineers [Supplementary Concept] OR Vater-Like Defects with Pulmonary Hypertension, Laryngeal Webs, and Growth Deficiency[Supplementary Concept] OR "Rowley-Rosenberg syndrome[Supplementary Concept] ) ORpulmonary artery hypertension[Title/Abstract] OR PAH[Title/Abstract] | 43761 |
| #3 | (endothelin receptor antagonists[MeSH Terms]) OR bosentan[Title/Abstract]) OR tracleer[Title/Abstract] | 5684 |
| #4 | #1 AND #2 AND #3 AND ("2000/01/01"[Date - Publication] : "2017/06/30"[Date - Publication]) | 18 |

**EMBASE (Ovid)**

| #1 | (‘Cost benefit analysis’/exp OR (cost*:ti,ab AND (benefit:ti,ab OR effectiveness:ti,ab OR utility:ti,ab) OR (marginal:ti,ab AND analys*:ti,ab) OR minimization OR minimisation) OR ((economic*:ti,ab OR pharmacoeconomic*:ti,ab) AND (analys*:ti,ab OR assessment*:ti,ab OR evaluat*:ti,ab OR implication:ti,ab OR health:ti,ab))) | 337042 |
| --- | --- | --- |
| #2 | ((‘Familial Primary Pulmonary Hypertension’.mp. OR ‘pulmonary hypertension’/ exp) OR (‘Persistent Fetal Circulation Syndrome’.mp. OR ‘persistent pulmonary hypertension’/ exp) OR (‘Pulmonary Arterial Hypertension’:ti,abOR ‘Hereditary Hemorrhagic Telangiectasia-Related’:ti,abOR ‘Dursun Syndrome’:ti,abOR ‘Pulmonary Hypertension, Primary, Autosomal Recessive’:ti,abOR ‘Cirrhosis, Familial, with Pulmonary Hypertension’:ti,abOR ‘Pulmonary edema of mountaineers’:ti,abOR ‘Vater-Like Defects with Pulmonary Hypertension, Laryngeal Webs, Growth Deficiency’:ti,abOR ‘Rowley-Rosenberg syndrome’:ti,ab. | 72320 |
| #3 | (‘endothelin receptor antagonist’/exp OR (Bosentan.mp. OR bosentan/ exp) OR Tracleer.mp.) | 15996 |
| #4 | #1 AND #2 AND #3AND ([2000-2017]/py) | 88 |

**Cochrane library**

| #1 | (‘Cost benefit analysis’/exp OR (cost*:ti,ab,kw AND (benefit:ti,ab,kw OR effectiveness:ti,ab,kw OR utility:ti,ab,kw) OR (marginal:ti,ab,kw AND analys*:ti,ab,kw) OR minimization OR minimisation) OR ((economic*:ti,ab,kw OR pharmacoeconomic*:ti,ab,kw) AND (analys*:ti,ab,kw OR assessment*:ti,ab,kw OR evaluat*:ti,ab,kw OR implication:ti,ab,kw OR health:ti,ab,kw))) | 54660 |
| --- | --- | --- |
| #2 | ((‘Familial Primary Pulmonary Hypertension’/expOR ‘pulmonary hypertension’/ exp OR ‘Persistent Fetal Circulation Syndrome’/exp) OR (‘Pulmonary Arterial Hypertension’:ti,ab,kwOR ‘Hereditary HemorrhagicTelangiectasia-Related’:ti,ab,kwOR ‘Dursun Syndrome’:ti,ab,kwOR ‘Pulmonary Hypertension, Primary, Autosomal Recessive’:ti,ab,kwOR ‘Cirrhosis, Familial, with Pulmonary Hypertension’:ti,ab,kwOR ‘Pulmonary edema of mountaineers’:ti,ab,kwOR ‘Vater-Like Defects with Pulmonary Hypertension, Laryngeal Webs, Growth Deficiency’:ti,ab,kwOR ‘Rowley-Rosenberg syndrome’:ti,ab,kw. | 1993 |
| #3 | (‘endothelin receptor antagonist’/exp OR bosentan:ti,ab,kw OR Tracleer:ti,ab,kw) | 468 |
| #4 | #1 AND #2 AND #3AND ([2000-2017]/py) | 11 |

**Proquest(EconLit)**

| #1 | su(Cost benefit analysis) OR ( (TI,AB(cost*) AND TI,AB(benefit OR effectiveness OR utility) OR TI,AB (marginal AND analys*) OR TI,AB (minimization OR minimisation))OR TI,AB(analys* OR assessment*OR evaluat* OR implication OR health) AND TI,AB(economic* OR pharmacoeconomic*)) | 258015 |
| --- | --- | --- |
| #2 | su((Familial Primary Pulmonary Hypertension) OR (Hypertension, Pulmonary) OR (Familial Primary Pulmonary ) OR (Persistent Fetal Circulation Syndrome))OR TI,AB(‘Pulmonary Arterial Hypertension’ OR ‘Hereditary Hemorrhagic Telangiectasia-Related’ OR ‘durbin Syndrome’ OR ‘Pulmonary Hypertension, Primary, Autosomal Recessive’OR ‘Cirrhosis, Familial, with Pulmonary Hypertension’ OR ‘Pulmonary edema of mountaineers’ OR ‘dater-Like Defects with Pulmonary Hypertension, Laryngeal Webs, Growth Deficiency’ OR ‘Rowley-Rosenberg syndrome’) | 57782 |
| #3 | SU(endothelin receptor antagonist) OR TI, AB(bosentan OR tracleer) | 6342 |
| #4 | #1 AND #2 AND #3AND ([2000-2017]/py) | 2 |

**Chinese database: China National Knowledge Infrastructure (CNKI)**

| Search strategy | Item found |
| --- | --- |
| SU=肺动脉高压 AND (SU=费用 OR SU=成本 OR SU=经济 OR SU=负担 OR SU=卫生支出 OR SU=卫生费用) AND (SU=波生坦 OR SU=全可利)  Publication Date : 2000-01-01 to 2017-06-30 | 10 |

**Chinese database: Wanfang Data**

| Search strategy | Item found |
| --- | --- |
| 主题:(肺动脉高压) * (主题:(费用) + 主题:(成本) + 主题:(经济) + 主题:(负担）+ 主题:(卫生支出) + 主题:(卫生费用)) * (主题:(波生坦) + 主题:(全可利))  Publication Date : 2000 to 2017 | 32 |

**Chinese database: the Chongqing VIP (CQVIP)**

| Search strategy | Item found |
| --- | --- |
| M=肺动脉高压* (M=费用+ M=成本+ M=经济+ M=负担+ M=卫生支出+ M=卫生费用) *(M=波生坦+ M=全可利)  Publication Date : 2000 to 2017 | 2 |
